# Bi‐Allelic *DSG1* Splice‐Site Variant Identified in a Family With Non‐Syndromic Striate Palmoplantar Keratoderma

**DOI:** 10.1111/1346-8138.17911

**Published:** 2025-08-29

**Authors:** Sohail Ahmed, Nicole Cesarato, Ye Li, Xing Xiong, Kifayat Ullah, Hammal Khan, Muhammad Javed Khan, Holger Thiele, Wasim Ahmad, Muhammad Sharif Hasni, Regina C. Betz

**Affiliations:** ^1^ Institute of Human Genetics Medical Faculty and University Hospital Bonn, University of Bonn Bonn Germany; ^2^ Institute of Biochemistry University of Balochistan Quetta Pakistan; ^3^ Department of Biochemistry, Faculty of Biological Sciences Quaid‐i‐Azam University Islamabad Pakistan; ^4^ Cologne Center for Genomics University of Cologne Cologne Germany

**Keywords:** biological variation, Desmoglein 1, exome sequencing, palmoplantar keratoderma, splicing

## Abstract

Hereditary palmoplantar keratoderma (PPK) involves hyperkeratosis of the palmoplantar skin and belongs to the palmoplantar epidermal differentiation disorders (pEDDs). One causal gene is Desmoglein 1 (*DSG1*), which encodes a protein crucial for epidermal integrity. Monoallelic *DSG1* variants cause mild, non‐syndromic PPK, whereas bi‐allelic *DSG1* variants typically cause syndromic PPK with severe additional clinical features (SAM syndrome). Here, we report the first detection of a homozygous *DSG1* variant in mild, non‐syndromic PPK. Pakistani siblings presented with striate PPK, characterized by deep palmar creases and plantar fissures only. Exome sequencing revealed the homozygous *DSG1* splice‐site variant c.685‐3T>A with familial cosegregation. In silico analyses indicated a low probability of exon 7 skipping. An exon‐trap assay confirmed splicing disruption, although some wild‐type (WT) transcripts were also detected. The partial retention of DSG1 WT transcripts may explain the mild phenotype. This finding highlights the phenotypic variability of *DSG1*‐related disorders (*DSG1*‐pEDD), related to residual DSG1 activity.

## 1 Introduction

Palmoplantar keratodermas (PPKs) are a heterogeneous group of acquired and congenital disorders of the palmoplantar skin involving epidermal hyperkeratotic thickening [1]. According to the recent classification proposed by Sprecher et al., PPK is categorized within the palmoplantar epidermal differentiation disorders (pEDDs), and each disorder is designated by listing the causative gene followed by the suffix ‘pEDD’ [2]. PPK can exhibit a range of keratinization patterns, including diffuse, focal, and striate forms. PPK may present as non‐syndromic, with disease confined to the palms and soles, or as syndromic, involving additional abnormalities such as nail dystrophy, dental anomalies, and/or dysfunction of other organs [1, 2]. The mode of inheritance varies according to the underlying genetic defect and may be either autosomal dominant or autosomal recessive [1, 2].

This study focuses on the genetic causes of non‐syndromic (mild) PPK, which involve pathogenic variants in genes encoding proteins such as keratins, gap junction components, and desmosomal proteins, which are critical for keratinization and structural integrity of the epidermis [1, 2]. One such gene implicated to date is Desmoglein 1 (*DSG1*), which encodes the desmosomal protein DSG1 and is responsible for *DSG1*‐pEDD [3].

In individuals with a heterozygous *DSG1* pathogenic variant leading to haploinsufficiency, the condition most commonly manifests as striate PPK in early childhood (MIM:148700). Importantly, the effects of heterozygous variants are confined to the skin, with no involvement of other organs or systems [3]. In contrast, individuals with bi‐allelic *DSG1* pathogenic variant(s) can develop a much more severe, systemic disorder known as SAM syndrome (MIM:615508) [4]. In this condition, the mutations lead to a complete or near‐complete loss of desmoglein 1 function.

Here, we report the genetic analysis of a consanguineous Pakistani family who presented with isolated striate PPK. Molecular genetic analysis identified a homozygous splice site variant in *DSG1*, and subsequent splicing analysis demonstrated partial impairment of normal splicing.

## 2 Case Report

**FIGURE 1:**
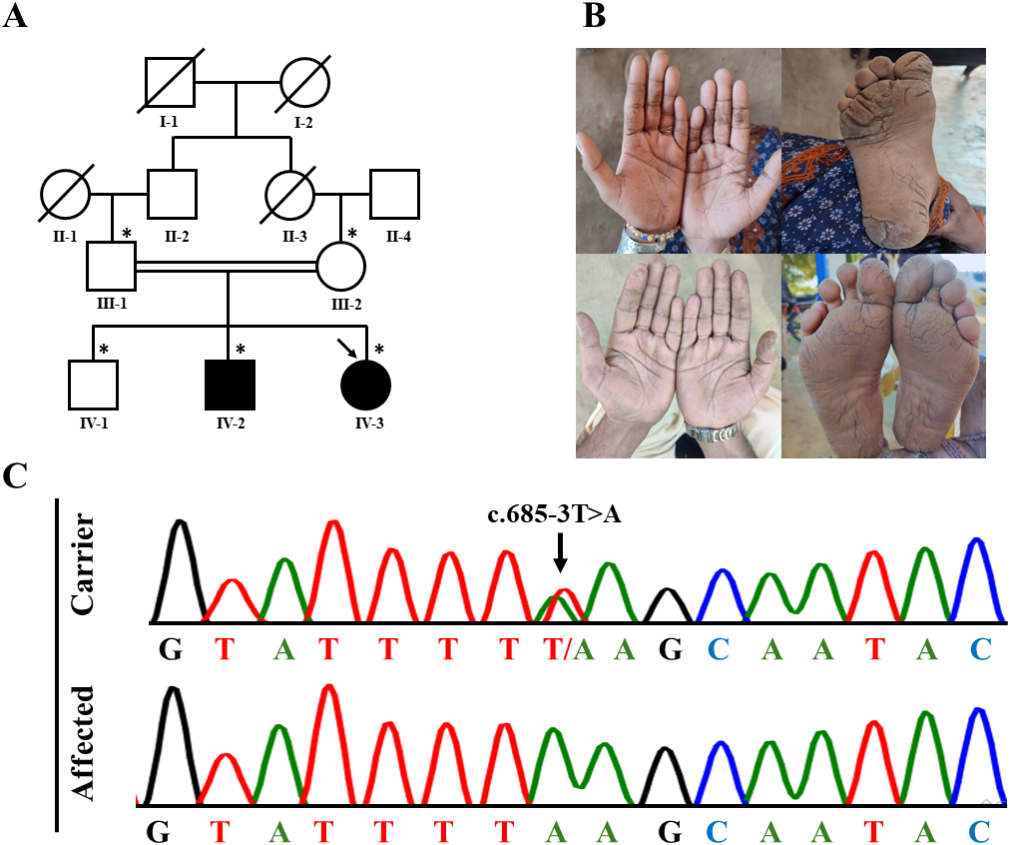
(A) Family pedigree. Males and females are represented by squares and circles, respectively. Filled symbols represent the affected individuals and the arrow indicates the sibling whose DNA was subjected to ES. The asterisk indicates individuals from whom DNA is available. (B) Clinical features. The upper panel shows individual IV‐3, a 22 year old female with mild palmar creases and deep plantar fissures, and the lower panel represents individual IV‐2, a 27‐year‐old male with milder palmoplantar hyperkeratosis. (C) Sequencing results. Chromatograms of the variant in a carrier and an affected individual.

Two male and female adult siblings presented with striate PPK on either palms and/or soles. The parents were first‐degree cousins. Sibling IV‐2 displayed mild palmoplantar creases since the age of 5. Starting at 4 years of age, sibling IV‐3 displayed milder palmar creases with deep plantar fissures, particularly on pressure points (Figure 1). In both siblings, nails, teeth, hair and other organs were normal. Both parents and another male sibling were unaffected, and there was no known family history of PPK.

Exome sequencing (ES) of individual IV‐3 was performed at the Cologne Center for Genomics (Cologne, Germany). This led to the identification of the homozygous splice acceptor‐site variant, c.685‐3T>A, in *DSG1*. Sanger sequencing confirmed both the variant in individual IV‐3 and its familial cosegregation. The variant is rare (allele frequency of 0.00009882 in gnomAD v.4.1.0) and is observed exclusively in South Asian individuals [5].

To assess the impact of the variant on *DSG1* splicing, in silico analyses were performed using the HSF Pro System [6], SpliceAI [7], and Pangolin [8]. Both SpliceAI and Pangolin predicted possible splicing changes with low probability: a loss of the nearby splice acceptor site (score SpliceAI: 0.25; score Pangolin: 0.3) and the activation of a novel acceptor site located 98 bp upstream (score SpliceAI: 0.56; score Pangolin: 0.29). In contrast, HSFpro predicted no significant splicing effect.

To validate these predictions, a genomic fragment of *DSG1* spanning from part of intron 5 to part of intron 8—thereby encompassing exons 6–8 as well as introns 6 and 7 and containing either the WT or the mutated sequences—was cloned into the Exontrap Cloning Vector (MoBiTec, Germany). The plasmids were separately transiently transfected in HaCaT cells. Twenty‐four hours post‐transfection, the RNA was extracted and reverse transcription was performed. The resulting cDNA was amplified using plasmid‐specific primers and subsequently sequenced.

**FIGURE 2:**
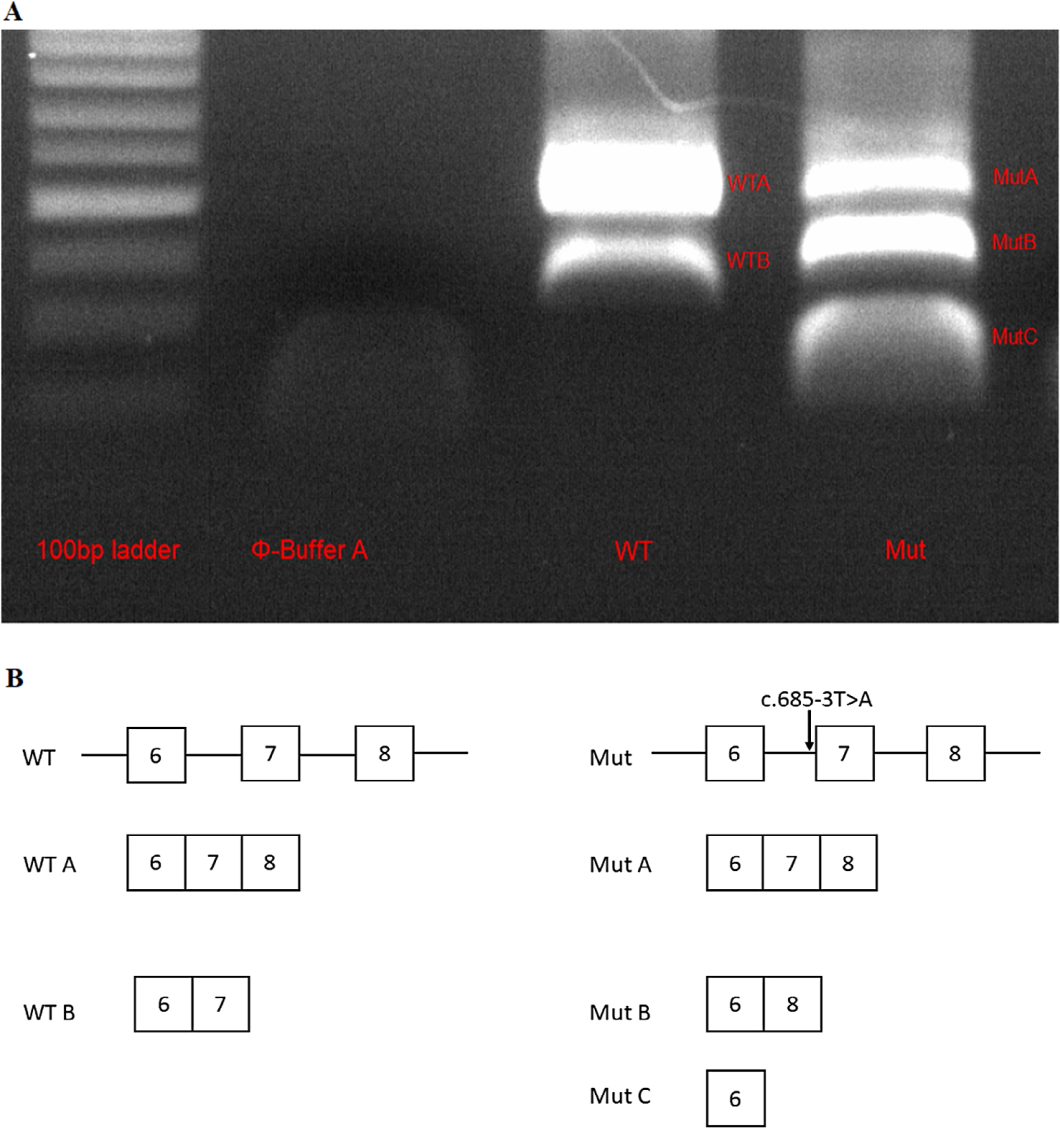
(A) Gel electrophoresis of WT and mutant *DSG1* transcripts generated by the Exontrap vector. (B) Schematic visualization of the splicing transcripts of *DSG1* produced by expression of the Exontrap vector containing exons 6, 7, and 8 of *DSG1*; the plasmids containing the wild‐type sequence and the variant are indicated by WT and MUT, respectively.

The plasmid containing the WT sequence produced two spliced isoforms: WT A, which includes exons 6, 7, and 8, and represents the canonical WT transcript; and WT B, which includes exons 6 and 7 only. Notably, the band corresponding to the canonical transcript was more prominent (Figure 2). In contrast, the plasmid containing the c.685‐3T>A variant generated three distinct splicing isoforms: MUT A, which includes exons 6, 7, and 8 and corresponds to the WT transcript; MUT B, which skips exon 7; and MUT C, which includes exon 6 only. These results indicate that the c.685‐3T>A variant has a mild effect on splicing and still allows the production of the WT transcript (MUT A). Interestingly, the intensities of the bands for MUT A and MUT B were comparable, suggesting that the variant impairs splicing in approximately 50% of the transcripts (Figure 2). MUT B and MUT C have the potential to lead to in‐frame deletions of 45 and 107 amino acids, respectively.

## 3 Discussion

The present report describes two siblings with striate PPK arising secondary to a homozygous splice variant in *DSG1* (c.685‐3T>A). To our knowledge, this is the first reported case of mild PPK secondary to a bi‐allelic *DSG1* variant.

Exontrap experiments revealed that, in ~50% of transcripts, this variant leads to exon 7 skipping, or to combined exon 7 and 8 skipping. Despite this, a fraction of WT transcripts is retained, and thus, we suggest that the impact on splicing is mild. While computational splicing programs predicted no splicing effect, SpliceAI and Pangolin identified an impaired splice acceptor site, albeit with low probability scores in reflection of the variant's mild effect. Interestingly, these tools also predicted activation of a cryptic splice site, which we could not confirm experimentally. This discrepancy could indicate a misprediction. Alternatively, it could stem from limitations in our in vitro setup, since no RNA was available from affected individuals. These findings reinforce the importance of the in vivo or in vitro validation of splicing variants.

DSG1 plays a role in epidermal adhesion and the maintenance of epidermal tissue integrity. Research suggests that monoallelic variants in *DSG1* cause mild PPK via haploinsufficiency [8]. Bi‐allelic variants in *DSG1* usually cause SAM syndrome with wide clinical heterogeneity [9]. Here, the reported siblings presented for the first time with isolated/mild PPK despite a homozygous *DSG1* variant.

The least severely affected bi‐allelic *DSG1* variant carrier described in the literature to date is an Egyptian individual who presented with PPK, skin fragility, elevated IgE levels, and no history of allergies [10]. Due to the rather mild clinical features, a clinical diagnosis of SAM syndrome was excluded. Interestingly, despite RNA analysis showing complete exon 14 skipping, DSG1 immunostaining of a skin biopsy from this individual revealed only a slight signal reduction.

On the basis of our findings and those of previous research, we propose that the observed variability in *DSG1*‐related phenotypes is linked to the residual activity of the DSG1 protein. We suggest that isolated PPK occurs when approximately 50% of functional DSG1 is lost, either through heterozygous loss‐of‐function variants or bi‐allelic splice variants that impair around 50% of transcripts. We propose that greater reductions in DSG1 activity result in additional cutaneous and extracutaneous symptoms, with SAM syndrome representing the most severe phenotype. This aligns with the observation that heterozygous *DSG1* variant carriers—mostly comprising parents of SAM patients—only exhibit a phenotype when the variant has a significant disruptive effect on protein function. Indeed, asymptomatic heterozygous *DSG1* variant carriers were detected in both the present family and the family of the aforementioned patient from Egypt, whereas heterozygous carriers of *DSG1* variants implicated in SAM syndrome usually display PPK.

In conclusion, the present report describes Pakistani siblings with mild PPK secondary to a homozygous *DSG1* splice variant with a mild effect on splicing. Our findings expand the mutational and phenotypic spectrum of *DSG1* variants and emphasize the correlation between phenotypic variability and residual DSG1 activity.

## Ethics Statement

All study procedures were performed in accordance with the principles of the Declaration of Helsinki and were approved by the Institutional Review Board (IRB) of Quaid‐i‐Azam University Islamabad (IRB/QAU/176), Pakistan. Written informed consent was obtained from the legal guardians of the affected siblings included in the study for both the conduct of the study and the presentation of data/photographs in publications.

## Conflicts of Interest

The authors declare no conflicts of interest.

## Data Availability

The data that support the findings of this study are available from the corresponding author upon reasonable request.
